# Automating Injury Severity Score Calculation From Free-Text Discharge Diagnoses Using Domain-Specific Retrieval-Augmented Large Language Models: Retrospective Validation Study

**DOI:** 10.2196/93922

**Published:** 2026-10-13

**Authors:** Ying-Jia Lin, Kuan-Yueh Fang, Chien-Hung Liao, Chi-Tung Cheng

**Affiliations:** 1 Department of Artificial Intelligence College of Intelligent Computing Chang Gung University Taoyuan Taiwan; 2 Artificial Intelligence Research Center Chang Gung University Taoyuan Taiwan; 3 School of Medicine Chang Gung University Taoyuan Taiwan; 4 Department of Surgery Division of Trauma and Emergency Surgery Chang Gung Memorial Hospital Taoyuan Taiwan

**Keywords:** Abbreviated Injury Scale, automatic ISS calculation, large language models, retrieval-augmented generation, trauma

## Abstract

**Background:**

Trauma registries are crucial for evaluating the prognosis of injuries and the quality of trauma care. However, the calculation of Injury Severity Score (ISS) remains a predominantly manual process, which is labor-intensive, potentially error-prone, and difficult to scale. Automating this process is a critical step toward enhancing the efficiency and quality of trauma registry data.

**Objective:**

This study conducts a feasibility test on the use of large language models (LLMs) to automatically calculate ISS from free-text discharge diagnoses. We specifically investigate the efficacy of retrieval-augmented generation (RAG) to enhance LLM accuracy by incorporating knowledge from the Abbreviated Injury Scale. We compare the performance of baseline LLMs against models augmented with both a standard RAG and a novel, domain-specific RAG framework.

**Methods:**

In this retrospective validation study, we used deidentified, free-text discharge diagnoses from trauma patients at Chang Gung Memorial Hospital (Linkou, Taiwan) in 2022. Against a physician-verified gold standard, we compared the performance of three systems: (1) baseline LLMs, (2) LLMs with standard RAG, and (3) LLMs with domain-specific RAG, for which the retriever was fine-tuned on trauma-related *ICD-10* (*International Statistical Classification of Diseases, Tenth Revision*) codes. Performance was evaluated on a physician-verified test set across exact ISS prediction, tolerance-based accuracy, and ISS severity categories.

**Results:**

Baseline state-of-the-art LLMs achieve modest exact-match (EM) accuracy, ranging from 47.3% to 64.7%. Standard RAG did not produce consistent gains (54.3% to 60.3%), whereas the domain-specific RAG substantially improved accuracy (62.7% to 75.3%). Performance gains were most pronounced in patients with moderate to severe injuries. GPT-4.1 with domain-specific RAG reached the highest EM accuracy (75.3%, 95% CI 66.1%-82.7%), compared with 70.0% for the deterministic International Classification of Diseases and R statistical software (ICDPIC-R) baseline. Tolerance-based and categorical analyses confirm substantial gains with the domain-specific RAG framework in clinically relevant ranges.

**Conclusions:**

This single-center feasibility study suggests that LLMs augmented with a domain-specific retriever can calculate the ISS from free-text discharge diagnoses with overall accuracy comparable to that of a deterministic coding tool, with the advantage concentrated in the most severe injuries. This approach may help alleviate the administrative burden of manual coding, enhance standardization, and improve the quality of trauma registry data. Future multi-institutional validation is needed to confirm these findings.

## Introduction

Trauma registries are pivotal for evaluating trauma care performance [1,2]. Among the elements in the trauma registry database, the calculation of Injury Severity Score (ISS) represents a crucial step in quantifying overall disease severity, especially in patients with multiple traumas [3]. However, the reliability of ISS is often challenged, since the calculation of ISS heavily depends on trauma registry coders. Although regular accuracy audits on random cases can mitigate this issue, the associated costs are often impractical for many institutions [4]. In addition, ISS coding remains a labor-intensive process, further limiting the efficiency of trauma registries and timely use in supporting clinical decision-making.

Previous studies have explored automated ISS calculation mainly through structured data mapping. For example, Abajas-Bustillo et al [5] applied a 2-step *ICD-9-CM* (*International Classification of Diseases, Ninth Revision, Clinical Modification*) to Abbreviated Injury Scale (AIS) conversion process, achieving high concordance for identifying major trauma cases but encountering mapping gaps and underestimation issues. Niemann et al [6] extended this approach to *ICD-10-GM* (*International Statistical Classification of Diseases and Related Health Problems, Tenth Revision, German Modification*) using a custom *ICD-10-CM* (*International Classification of Diseases, Tenth Revision, Clinical Modification*) mapping pipeline with International Classification of Diseases and R statistical software (ICDPIC-R), showing equivalence to manual coding only in certain ISS ranges. Beyond code mapping, Yang et al [7] used ultraearly physiologic data with machine learning to estimate ISS, enabling rapid mortality risk assessment. More recently, Gao et al [8] combined clinical text with structured electronic health record data to stratify trauma injury severity across multiple body regions using multimodal machine learning models. However, these approaches rely on structured diagnostic codes, physiologic measurements, or multimodal data, leaving the potential for fully automated exact ISS calculation directly from unstructured clinical text underexplored.

The rapid development of large language models (LLMs) offers new opportunities to accelerate medical data processing. These models have shown increasing capability in tasks such as information extraction from unstructured clinical notes and the generation of decision support aids [9-11]. Chan et al [12] further demonstrated the feasibility of LLM-assisted ISS calculation through an interactive, clinician-assisted workflow using a web-based LLM service. However, the performance of LLMs under a standardized automated protocol without iterative clinician guidance remains unclear. Recent research on automated medical coding also suggests that general-purpose LLMs may lack the specialized knowledge required for standardized clinical coding. Hou et al [13] showed that incorporating domain-specific *ICD-10* (*International Statistical Classification of Diseases, Tenth Revision*) knowledge through model fine-tuning substantially improved automated medical coding performance. Furthermore, retrieval-augmented generation (RAG) [14] enhances LLMs by querying external knowledge bases, thereby grounding outputs in verifiable information and improving accuracy. While numerous studies have demonstrated the potential of LLMs in health care, their application to standardized and automated ISS calculation with explicit AIS knowledge retrieval remains underexplored. This study, therefore, applies LLMs to streamline the trauma registry workflow by investigating their feasibility for generating ISS scores directly from unstructured discharge diagnoses. We aim to address the following research question: Can LLMs, with or without RAG, accurately calculate ISS from unstructured discharge diagnoses, and does a domain-specific retriever improve RAG performance?

The primary contributions of this study are as follows:

1. We demonstrate the feasibility of using LLMs to directly estimate ISS from unstructured discharge diagnoses.
2. We develop and evaluate a domain-specific retriever, fine-tuned on trauma-related *ICD-10* codes, to improve the accuracy of RAG-based ISS calculation.
3. A comprehensive performance analysis is conducted across multiple metrics, including exact-match (EM) accuracy, tolerance-based scoring, and categorical classification, to provide a thorough clinical evaluation.

## Methods

### Overview

Our methodology for automated ISS calculation is based on a RAG framework, illustrated in Figure 1. The framework operates in two sequential stages: (1) retrieval—a domain-specific retriever identifies the most semantically relevant injury descriptions from the AIS 1998 knowledge base based on the input discharge diagnosis. This stage incorporates a reference expansion (RefExp) step to enhance recall by augmenting the results with injuries that share the same anatomical prefix (defined by the first 3 words in AIS descriptions). (2) Generation—an LLM, serving as the generator, synthesizes the retrieved AIS descriptions and the original discharge diagnosis to calculate the final ISS.

**Figure 1:**
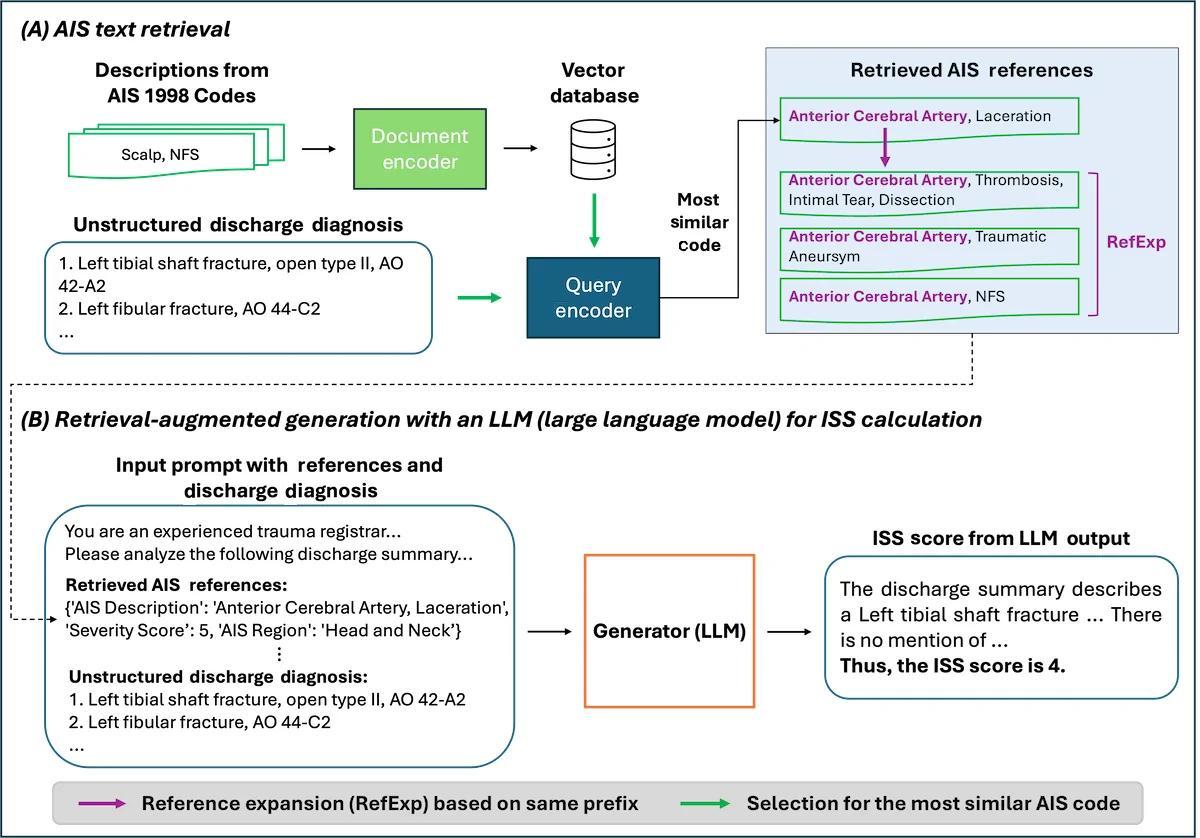
Overview of the retrieval-augmented generation framework evaluated in this retrospective validation study for automated Injury Severity Score (ISS) calculation using deidentified free-text discharge diagnoses from trauma patients treated at Chang Gung Memorial Hospital, Linkou, Taiwan, in 2022. (A) Relevant Abbreviated Injury Scale (AIS) 1998 injury descriptions are retrieved from a vector database using semantic similarity, with reference expansion (RefExp) to include injuries sharing the same anatomical prefix, reducing the risk of missing related injuries in the same body region. (B) The retrieved references and discharge diagnosis are provided to a large language model (LLM), which calculates the ISS for the trauma registry entry. NFS: not further specified.

### Ethical Considerations

This retrospective study was reviewed and approved by the institutional research ethics board (approval number 202500438B0) and was conducted in accordance with the Declaration of Helsinki. The requirement for informed consent was waived by the ethics board because the study involved the retrospective analysis of existing medical records. All electronic medical records were deidentified before analysis, and no information that could directly identify individual patients was available to the research team. No participants were recruited, contacted, or compensated for this study. The manuscript and supplementary materials contain no identifiable participant images or personal identifiers.

### Datasets

#### Data Collection and Preprocessing

We obtained the deidentified 2022 electronic medical records from the trauma department of Chang Gung Memorial Hospital, a level I trauma center in Taiwan. The raw dataset comprised 4392 cases, where each record included free-text discharge diagnoses, *ICD-10-CM* codes, AIS scores (1998 version, the current standard in Taiwan’s National Health Insurance System), and an ISS score. To construct a test set suitable for evaluating AIS identification and ISS calculation solely from free-text discharge diagnoses, we set aside 300 cases randomly selected through stratified random sampling with proportional allocation across 5 injury-severity strata (ISS 0-9, 10-16, 17-25, 26-36, and 37-75), so that the reviewed sample preserved the severity distribution of the full cohort. For performance reporting, we adopted the 4 standard ISS severity categories (mild 1-8, moderate 9-15, severe 16-24, and profound 25-75) following Javali et al [15] and Bolorunduro et al [16].

A physician reviewed cases from this pool of 300 cases, assessing whether each discharge diagnosis contained sufficient clinical detail to derive the corresponding AIS codes and ISS without consulting *ICD-10-CM* codes, imaging findings, or other diagnostic information. Our target was a test set of 100 physician-verified cases that preserved the severity distribution of the full cohort. A total of 90 cases were excluded because they could not be reliably annotated from the discharge diagnosis text alone: the text lacked sufficient detail, could not be interpreted without reference to other diagnostic fields, or was inconsistent with the recorded diagnosis codes. Review continued until the target had been met. Cases not reached by that point were neither confirmed as usable nor excluded. Because the highest-severity strata are rare in the source population, the usable cases obtained from the 300-case pool proved insufficient in the profound range (ISS 25-75) to meet this target. Four additional profound cases were therefore sampled at random from a portion of the registry that had been held out from retriever training (described in the next section) and were annotated under the identical procedure. The final physician-verified test set comprised 100 cases—96 from the stratified review pool and 4 supplementary profound cases—and its severity distribution differed from that of the full cohort by no more than 1.3 percentage points in any of the 5 strata (test set vs cohort: 70/100, 70% vs 3018/4392, 68.7%; 12/100, 12% vs 552/4392, 12.6%; 12/100, 12% vs 569/4392, 13%; 4/100, 4% vs 184/4392, 4.2%; and 2/100, 2% vs 69/4392, 1.6%). Table 1 reports the demographic and injury characteristics of the test set, stratified by the 4 ISS severity categories defined above.

**Table 1:** Characteristics of the physician-verified held-out test set of 100 trauma cases from Chang Gung Memorial Hospital, Linkou, Taiwan, in 2022, stratified by Injury Severity Score (ISS) category: mild (ISS 1-8), moderate (ISS 9-15), severe (ISS 16-24), or profound (ISS 25-75). Characteristics include patient age and sex, mechanism of injury (blunt, penetrating, or not documented in the registry), the length of the free-text discharge diagnosis (number of whitespace-delimited words), and the average number of physician-verified Abbreviated Injury Scale (AIS) codes per case.

Characteristic		Mild (ISS 1-8)	Moderate (ISS 9-15)	Severe (ISS 16-24)	Profound (ISS 25-75)	Total
Cases, n		45	28	13	14	100
Age (years), mean (SD)		48.4 (21.2)	56.1 (28.0)	51.2 (20.7)	58.1 (19.7)	52.3 (23.1)
Male sex, n (%)		24 (53)	17 (61)	8 (62)	11 (79)	60 (60)
**Mechanism of injury, n**						
	Blunt	39	27	13	14	93
	Penetrating	4	0	0	0	4
	Not documented	2	1	0	0	3
Discharge diagnosis length (words), mean (SD)		23.3 (12.7)	31.9 (22.2)	25.9 (14.4)	65.4 (37.6)	31.9 (24.8)
Number of AIS codes, mean (SD)		1.29	1.56	1.92	2.50	1.61

#### Retriever Training Set Construction

Since our dataset lacked direct AIS annotations for specific text spans, we adopted a proxy task strategy to train the retriever. We used trauma-related *ICD-10-CM* codes (S00 to T75) and their official descriptions to create training pairs, allowing the retriever to learn semantic alignments between clinical narratives and structured injury codes. At this phase, 8 records with no *ICD* (*International Classification of Diseases*) codes were removed from the 4092 cases. To mitigate the risk of overfitting the retriever on *ICD-10-CM* descriptions, we randomly selected 70% (2859 discharge diagnoses) of the records from the pool of 4084 cases for training rather than using all available cases. The remaining 1225 (30%) records were not used for training or hyperparameter tuning. The 4 supplementary test cases (mentioned in the previous section) were drawn from the held-out portion of this pool. No case in the final test set appears in the retriever’s training data. As a single discharge diagnosis may contain multiple *ICD-10* codes, this process yielded 7570 training examples in total. The system’s final performance was evaluated exclusively on a held-out test set of 100 discharge diagnoses. These cases were annotated by a physician, with a subset independently reviewed by a senior trauma specialist for quality assurance.

### Using LLMs for ISS Calculation

#### Prompt Design

In our prompts, we instructed an LLM to take the role of a senior trauma registrar. We then guided an LLM to extract the AIS codes based on the AIS 1998 code set and explicitly described how to calculate the ISS score from the extracted AIS codes. In addition to the guidance on ISS calculation, we also provided 2 simple examples to enhance the LLM’s understanding of ISS calculation. The prompts also included requirements to mention explanations or uncertainties to improve performance via reasoning [17] and to provide more transparent responses. Each model was instructed to return its analysis in JSON format, containing a required “explanation” field describing how the AIS scores and ISS were derived, a required “ISS” field holding the calculated score, and optional “limitations” and “uncertainties” fields. The final ISS used in all analyses was taken from the “ISS” field of each response; in the few cases where a model returned this field as an arithmetic expression rather than a single integer, the resulting value of that expression was used, and the ISS was never read from the “explanation” text. The same base prompt was used for all evaluated LLMs and experimental settings, with the retrieved AIS entries inserted before the discharge diagnosis in the RAG settings. The prompts used in this study are provided in section A in Multimedia Appendix 1.

#### RAG

While LLMs possess broad knowledge, they often lack specialized information such as the AIS 1998 code set. When prompted directly, these models exhibit a tendency to hallucinate or generate factually incorrect AIS codes, thereby making wrong ISS calculations. To mitigate this, we used a RAG framework to ground the model’s responses in an authoritative knowledge source. Our RAG architecture consists of 2 core components: a retriever and a generator. The retriever searches a vector database built from the AIS 1998 dictionary, which contains 1366 entries. Each entry has a 6-digit AIS predot code, a free-text injury description, an AIS severity value, and an ISS body region. For example, entry 150200 has the description “Skull, Fracture, Base,” a severity of 3, and the body region head or neck. Retrieval is performed over the description field. For each input discharge diagnosis, the retriever returns the top-*k* most similar descriptions (*k*=5 in this study). These retrieved entries, along with their severity scores and body regions, are then integrated with the original prompt (Table A.1 in Multimedia Appendix 1) for the inputs to the generator (the LLM). This approach transforms the generator’s task from open-ended severity estimation to a more constrained calculation based on the provided AIS codes.

#### Training the Retriever for RAG

Given the absence of ground-truth AIS annotations for our free-text data (as described in the “Datasets” section), we trained the retriever using a proxy task based on trauma-related *ICD-10-CM* codes (S00-T75). Next, we built the retriever in a biencoder architecture [14,18]. Both encoders were initialized from MedCPT [18]. Similar to Dense Passage Retrieval [19], we used a contrastive loss function to train the retriever. The training details are provided in section B.1 in Multimedia Appendix 1.

This proxy task is motivated by the substantial semantic overlap between *ICD-10-CM* trauma code descriptions and AIS injury descriptions, as both systems characterize injuries by anatomical location, injury type, and clinical severity. While the 2 coding systems differ in granularity—AIS descriptions encode finer morphological and severity-specific distinctions—the shared lexical and conceptual structure provides a reasonable training signal for learning clinically relevant embeddings in the absence of ground-truth AIS annotations.

#### RefExp

To further enhance the retriever’s effectiveness, we introduced a RefExp step after retrieving the top-*k* AIS descriptions. This mechanism augmented the top-*k* retrieved results with additional AIS descriptions sharing the same anatomical prefix. In this study, a 3-word prefix was selected as it provides a practical balance: shorter prefixes (eg, 2 words) tend to match overly broad anatomical categories, introducing excessive irrelevant entries, while longer prefixes (eg, 4 words) are too restrictive and fail to capture closely related injury variants.

#### Baselines

We evaluated the state-of-the-art LLMs at the time of writing, including GPT-4.1 [20], Gemini 2.5 Pro [21], Claude 4.5 Sonnet [22], and Llama 4 Maverick [23]. To minimize bias, the same prompt design was applied across all the LLMs. To ensure stability, each experiment was repeated 3 times. Although temperature was set to 0, inference through the commercial APIs is not strictly deterministic, and the 3 repetitions quantify this residual run-to-run variability. We denote the prompting-only setting without RAG as original, which serves as the baseline for comparison. For RAG integration, we adopted the pretrained MedCPT retriever [18] in the standard RAG setting, while we used our trained retriever in the domain-specific RAG (DS-RAG). Implementation details and the exact model names used in this study are provided in section B in Multimedia Appendix 1.

As a deterministic reference, we additionally computed ISS with ICDPIC-R [24] from the *ICD-10-CM* codes of the same 100 test cases, evaluating all 6 mapping methods and reporting the best-performing one (gem_max).

#### Evaluation Metrics

Model performance was evaluated on the held-out test set using 4 complementary metrics, with all results reported as the mean and SD over 3 runs.

##### EM Accuracy

A prediction was considered correct only if the predicted ISS was numerically identical to the physician-verified ISS.

##### Tolerance-Based Accuracy

Because the clinical distinction between adjacent ISS values is sometimes marginal (eg, an ISS of 16 vs 17, both within the “severe” category), we introduced a dual-condition tolerance criterion, where a prediction is accepted if both conditions below are satisfied:



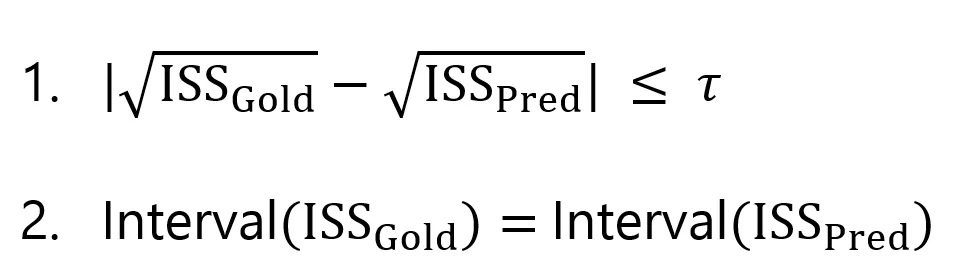



We set *τ*=1 for mild injuries (ISS≤8) and *τ*=0.5 for ISS≥9. The threshold was defined on the absolute difference between the square roots of the predicted (ISS_Pred_) and gold-standard ISS (ISS_Gold_), with predictions further required to remain in the same severity interval to avoid cross-category errors. More details and examples with thresholds are in section D in Multimedia Appendix 1.

##### Severity-Category Classification

To reflect clinical decision-making, which often relies on stratified severity categories rather than exact scores, we reformulated ISS calculation as a 4-class classification task over the standard ISS intervals: mild (1-8), moderate (9-15), severe (16-24), and profound (25-75) [15,16]. Performance was measured by accuracy and the macroaveraged *F*_1_-score to mitigate the impact of class imbalance.

##### Retrieval Quality

To evaluate the retrieval component independently of the generation stage, we compared the retrieved AIS descriptions against the physician-annotated AIS codes for each test case. Let *G_i_* denote the set of physician-verified AIS codes for case *i*, and *R_i_* the set of unique AIS codes retrieved for that case (the top-*k* results, together with the entries added by RefExp where applicable). A retrieved code was counted as correct only if it exactly matched a physician-verified AIS 1998 code. Per-case precision and recall were computed as



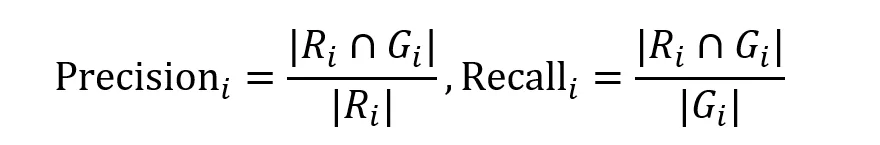



and macroaveraged precision and recall were obtained by averaging these values over the 100 test cases. Because the retriever is deterministic, retrieval metrics are reported as single values without SDs.

#### Statistical Analysis

To assess whether the performance differences among the original, standard RAG, and DS-RAG settings were statistically significant, we conducted a McNemar test on the predictions of the best-performing model, GPT-4.1. For each of the 100 test cases, per-case correctness was defined by EM accuracy (see “Evaluation Metrics” section). Because each setting was run 3 times, the 3 runs were first consolidated into a single per-case outcome by majority vote (ie, a case was counted as correct if at least 2 of the 3 runs were correct), yielding paired binary outcomes suitable for the McNemar test. We used the exact (binomial) 2-sided version of the test rather than the chi-square approximation, as it remains valid regardless of the number of discordant pairs. The same test was applied to compare GPT-4.1 with DS-RAG against the ICDPIC-R baseline [24] on the same 100 cases. A 2-sided *P* value of <.05 was considered statistically significant.

In addition, 95% CIs for the EM accuracy were calculated using the Wilson score interval for a binomial proportion (n=100), which, unlike the standard normal approximation, remains valid for proportions close to 0 or 1. Intervals are quoted in the text for the principal comparisons; intervals for all model and setting combinations are provided in section E in Multimedia Appendix 1. Because all settings were evaluated on the same 100 cases, the significance of between-setting differences was assessed using the paired McNemar test rather than by the overlap of these CIs.

## Results

### Model Accuracy Comparison

We present the results of the LLMs on the ISS calculation task in Figure 2. In the original setting (without RAG), models achieved modest EM accuracy. Among the LLMs, Claude 4.5 Sonnet performed the best (64.7%), followed by Gemini 2.5 Pro (59.0%) and GPT-4.1 (57%), with Llama 4 Maverick the lowest (47.3%). After integrating the standard RAG, the changes were small and inconsistent. GPT-4.1 rose only marginally, from 57.0% to 58.7%, as did Gemini 2.5 Pro, from 59.0% to 60.3%; Llama 4 Maverick improved more clearly, from 47.3% to 55.0%. Claude 4.5 Sonnet, by contrast, declined by 10.3 percentage points, from 64.7% to 54.3%, falling from the strongest baseline model to among the weakest. Retrieval with a general-purpose biomedical retriever is therefore not reliably beneficial and can actively degrade an already strong baseline. The largest performance gains were observed with DS-RAG, which uses the retriever fine-tuned on trauma codes. Under this configuration, all models demonstrated improvements. Notably, GPT-4.1 with DS-RAG achieved the highest EM accuracy of 75.3% (95% CI 66.1%-82.7%), an increase of 18.3 percentage points over its own baseline of 57.0% (95% CI 47.2%-66.3%). It also exceeded the ICDPIC-R baseline (70.0%) by 5.3 points, although this paired difference did not reach statistical significance (15 vs 9 discordant pairs; exact McNemar *P*=.31). Gemini 2.5 Pro and Llama 4 Maverick also improved substantially, by 6.3 and 15.3 percentage points, respectively. The gain for Claude 4.5 Sonnet was much smaller (64.7% to 66.3%; +1.7 percentage points); we examine this exception in the severity-stratified analysis below.

To further validate these findings, we conducted a McNemar test on the predictions of our best-performing model, GPT-4.1. DS-RAG significantly outperformed the original setting (25 vs 7 discordant pairs; *P*=.002) and also significantly outperformed standard RAG (20 vs 2 discordant pairs; *P*<.001), whereas the original and standard RAG settings did not differ (14 vs 14 discordant pairs; *P*>.99). This underscores the necessity of a domain-specific retriever for handling the complexities of trauma discharge diagnoses. We note that the CIs reported above reflect the sampling uncertainty of the 100-case test set, whereas the error bars in Figures 2-4 reflect run-to-run variability across the 3 repetitions. CIs for all models and settings are reported in Table E.1 in Multimedia Appendix 1.

**Figure 2:**
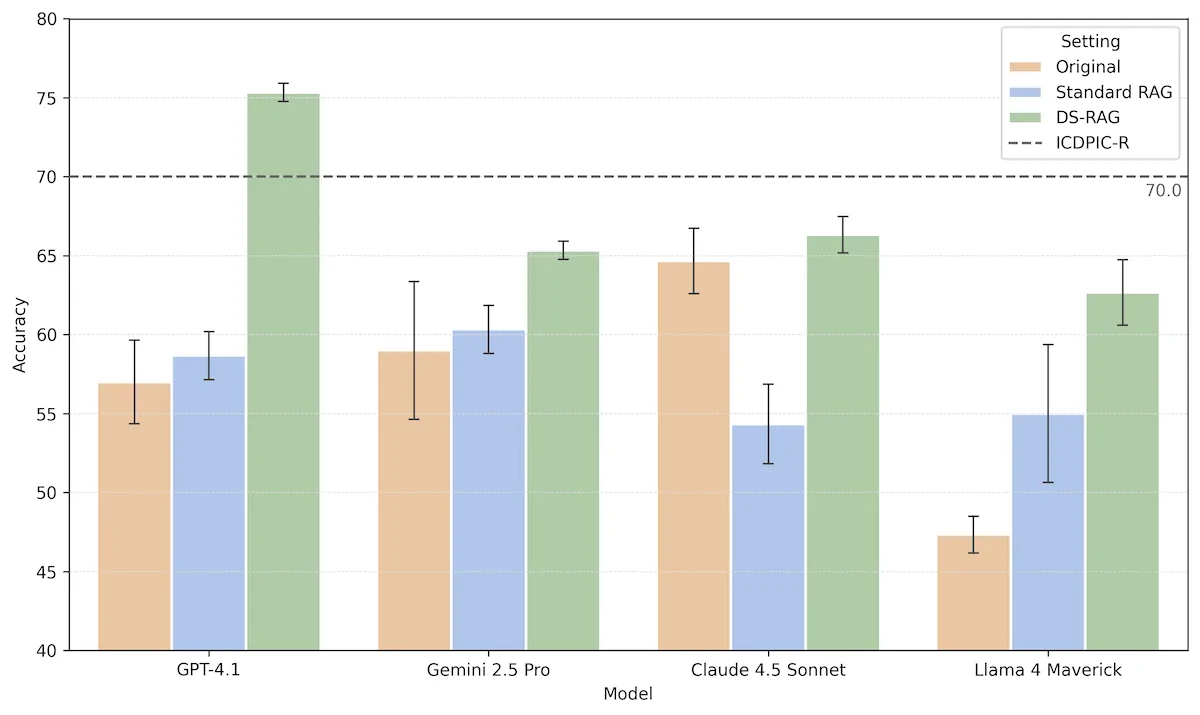
Exact-match accuracy of 4 large language models for automated Injury Severity Score calculation in a physician-verified test set of 100 trauma cases from Chang Gung Memorial Hospital, Linkou, Taiwan, in 2022. Exact match was defined as equality between the predicted ISS and physician-verified ISS. The 3 experimental settings were original (prompting only), standard retrieval-augmented generation (RAG) using the pretrained MedCPT retriever [18], and domain-specific RAG (DS-RAG) using a retriever fine-tuned on trauma-related International Classification of Diseases, Tenth Revision, Clinical Modification data (proposed). The horizontal dashed line indicates the International Classification of Diseases and R statistical software (ICDPIC-R; gem_max) baseline [24]. Bars represent mean accuracy, and error bars represent the SD over 3 runs.

**Figure 3:**
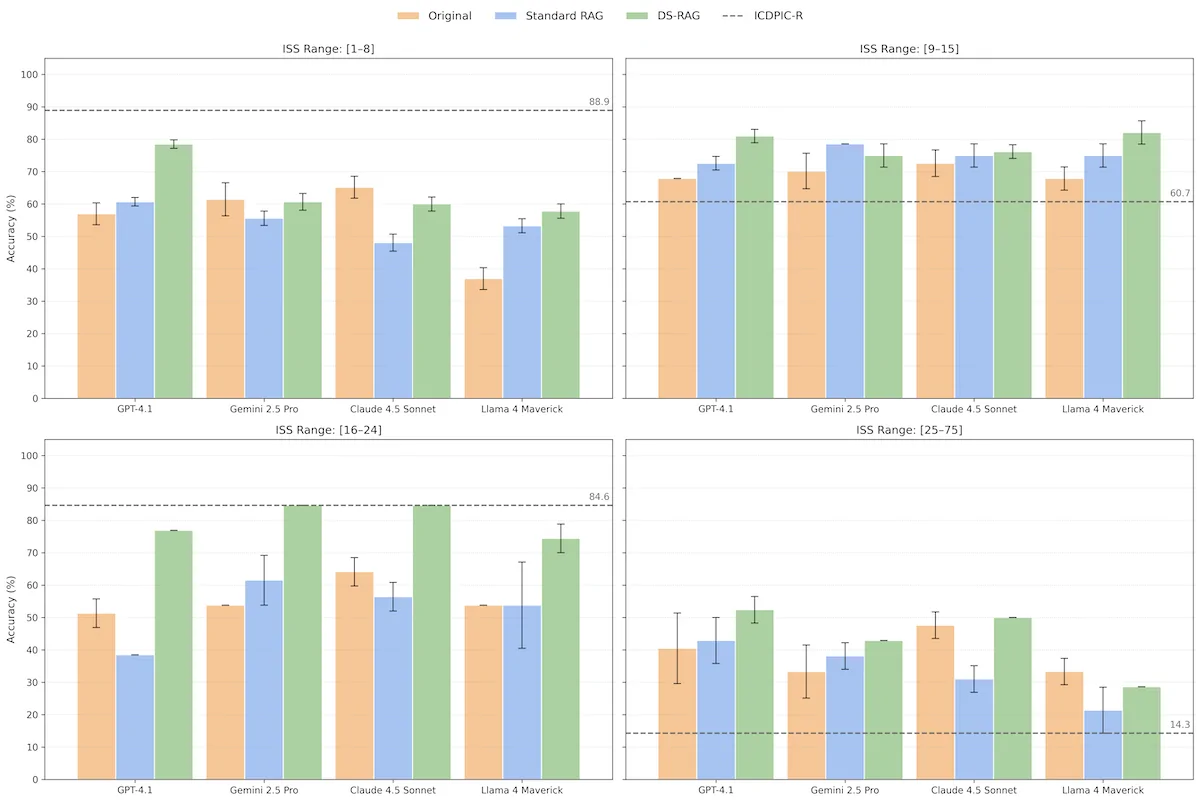
Exact-match accuracy of 4 large language models for automated Injury Severity Score (ISS) calculation, stratified by injury severity, in our physician-verified test set of 100 trauma cases from Chang Gung Memorial Hospital, Linkou, Taiwan, in 2022. The severity groups were mild (ISS 1-8; n=45), moderate (ISS 9-15; n=28), severe (ISS 16-24; n=13), and profound (ISS 25-75; n=14). Exact match was defined as equality between the predicted and physician-verified ISS. The experimental settings were original (prompting only), standard retrieval-augmented generation (RAG) using the pretrained MedCPT retriever [18], and domain-specific retrieval-augmented generation (DS-RAG) using a retriever fine-tuned on trauma-related International Classification of Diseases, Tenth Revision, Clinical Modification data (proposed). The horizontal dashed line indicates the International Classification of Diseases and R statistical software (ICDPIC-R; gem_max) baseline [24]. Bars represent mean accuracy, and error bars represent the SD over 3 runs.

**Figure 4:**
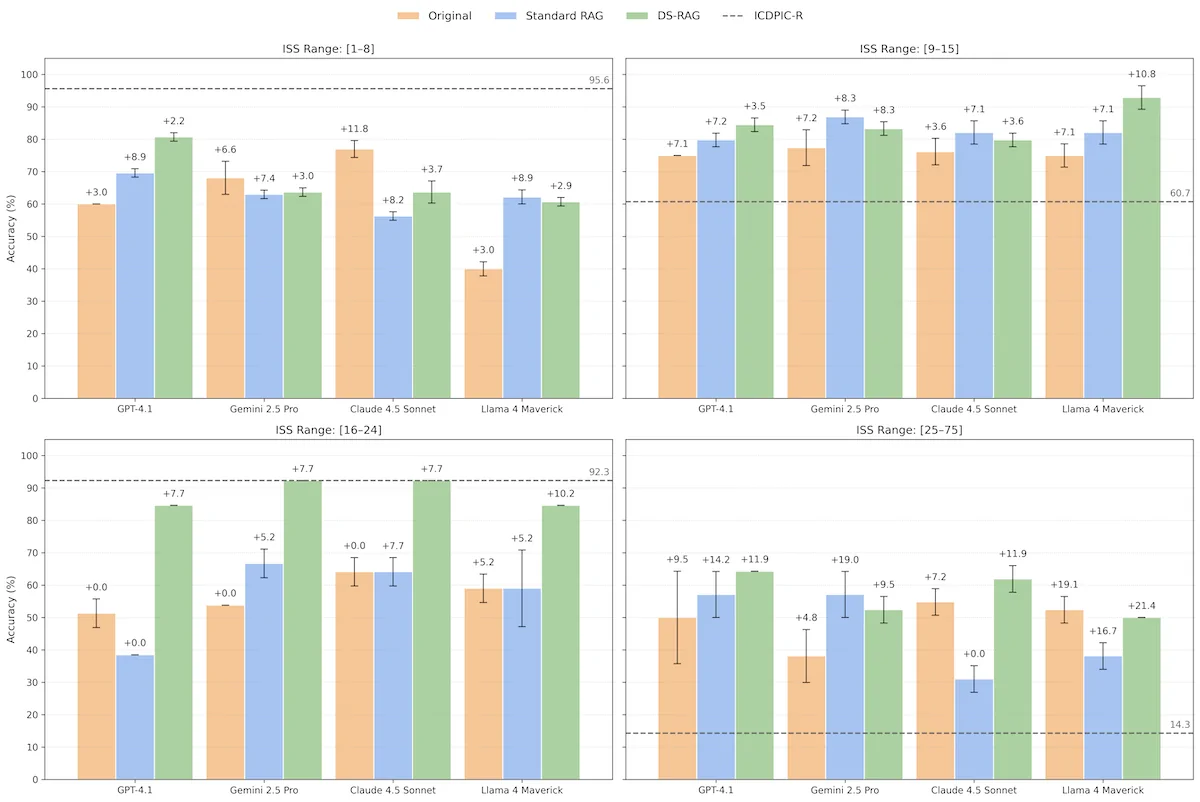
Tolerance-based accuracy of 4 large language models (LLMs) for automated Injury Severity Score (ISS) calculation, stratified by injury severity, in our physician-verified test set of 100 trauma cases from Chang Gung Memorial Hospital, Linkou, Taiwan, in 2022. The severity groups were mild (ISS 1-8; n=45), moderate (ISS 9-15; n=28), severe (ISS 16-24; n=13), and profound (ISS 25-75; n=14). A prediction was considered correct when the absolute difference between the square roots of the predicted and physician-verified ISS was within the predefined threshold (τ=1 for ISS≤8; τ=0.5 for ISS≥9) and both scores remained in the same severity group. The +N labels indicate the absolute percentage-point increase in tolerance-based accuracy relative to the corresponding exact-match accuracy in Figure 3 for the same model, experimental setting, and ISS group. The horizontal dashed line indicates the International Classification of Diseases and R statistical software (ICDPIC-R; gem_max) baseline [24]. Bars represent mean accuracy, and error bars represent the SD over 3 runs.

### Performance Analysis in Different ISS Ranges

To gain a more granular understanding of model performance, we stratified the EM accuracy analysis by standard ISS severity ranges: mild (ISS 1-8), moderate (9-15), severe (16-24), and profound (25-75) [15,16]. As shown in Figure 3, DS-RAG substantially improved EM accuracy in the mild injury range for GPT-4.1 and Llama 4 Maverick, whereas Gemini 2.5 Pro was essentially unchanged and Claude 4.5 Sonnet declined. This mild range decline largely accounts for Claude’s overall exception noted above, as it offset the model’s gains in the other severity ranges. The deterministic ICDPIC-R baseline attained the highest mild-range accuracy of any configuration (88.9%; dashed line in Figure 3), exceeding even the best LLM setting (GPT-4.1 with DS-RAG, 78.5%).

For the moderate (9-15) and severe (16-24) injury intervals, DS-RAG also outperformed the original setting for most models. Notably, in the severe interval of 16-24, though the difficulty of the ISS calculation task increases, all models with the DS-RAG setting still achieve better performance than the original setting, while the standard RAG setting does not show consistent improvement on GPT-4.1 and Claude 4.5 Sonnet. This indicates that the trained retriever with *ICD-10-CM* data can provide more relevant AIS-related context to the LLMs and enable them to better handle the severe injury cases. ICDPIC-R showed the opposite pattern across these 2 intervals: its accuracy fell to 60.7% in the moderate range, below every DS-RAG configuration, but reached 84.6% in the severe range, matching the best LLM configuration (Gemini 2.5 Pro with DS-RAG). We note that Llama 4 Maverick showed a relatively large SD across the 3 runs in the severe subgroup. This variability may reflect run-to-run differences in model predictions amplified by the small subgroup size (n=13).

For the profound injury range (25-75), the performance of all models dropped substantially. In this range, most models struggled to reach 50% EM accuracy, though GPT-4.1 with DS-RAG performed the best (52.4%), followed by Claude 4.5 Sonnet with DS-RAG (50.0%; Figure 3). ICDPIC-R performed the worst in this range. This suggests that the ISS calculation task becomes extremely challenging as the severity of injuries greatly increases, with profound cases involving the highest average number of AIS codes among the 4 ranges (Table 1). DS-RAG improved profound accuracy for GPT-4.1, Gemini 2.5 Pro, and Claude 4.5 Sonnet, but not for Llama 4 Maverick. Standard RAG was less reliable in this range, reducing accuracy for both Claude 4.5 Sonnet and Llama 4 Maverick relative to their baselines. We note that the profound subgroup contains only 14 cases, so a single case corresponds to 7.1 percentage points.

### Performance Analysis in Different ISS Ranges With Tolerance Threshold

While EM accuracy provides a strict assessment of model performance, we evaluated the models using the dual-condition tolerance criterion defined in the Evaluation Metrics section.

The tolerance analysis in Figure 4 reveals several important insights. The +N annotations in Figure 4 indicate the percentage-point increase in tolerance-based accuracy relative to the corresponding EM accuracy in Figure 3 for the same model, RAG setting, and ISS range. First, we discovered that accuracy scores of the models increased for most of the ISS ranges, particularly for the profound ISS range. These results indicate that while LLMs might not achieve perfect numerical precision in ISS prediction, many of their estimates were clinically reasonable approximations that fell within acceptable tolerance boundaries. ICDPIC-R showed the opposite behavior: it gained under the tolerance criterion only in the mild (95.6%) and severe (92.3%) ranges, with no gain at all in the moderate (60.7%) or profound (14.3%) ranges—its errors there were not near-misses but gross deviations. Second, the smallest increases from EM to tolerance-based accuracy were observed in the 16-24 range (mean +4.7, SD 3.8 points), where several models under the original setting showed no gain at all. This may be attributed to the complex interplay of multiple moderate injuries that characterize this ISS interval. Most notably, DS-RAG demonstrated superior performance under the proposed tolerance criterion, suggesting that improved contextual information not only enhanced accuracy but also led to more clinically meaningful approximations. This finding supports the practical applicability of RAG-enhanced LLMs in clinical decision-making scenarios where approximate severity assessment within reasonable bounds may be sufficient for initial triage and treatment planning.

### ISS Range Classification Performance

While precise, numerical ISS values are standard for registry data, clinical decision-making and prognostic assessments often rely on stratified injury severity categories rather than exact integer scores. To further analyze the difference between LLMs’ predictions and ground-truth ISS scores, we reformulated ISS calculation as a 4-class classification task over ISS intervals (1-8, 9-15, 16-24, and 25-75). In this task, a prediction is considered correct if it falls into the correct range (eg, a predicted ISS of 16 for a gold-standard ISS of 24 is correct for 16-24). In addition to accuracy, we evaluated performance using *F*_1_-score, which considers both recall and precision while mitigating the impact of class imbalance in our test dataset.

As shown in Table 2, DS-RAG yielded consistent performance gains for all models in both accuracy and *F*_1_-score. GPT-4.1 and Llama 4 achieved the largest *F*_1_-score improvements (+16.71 and +12.13), and GPT-4.1 remained the most accurate model (84.00% accuracy). In addition, standard RAG also improved accuracy and *F*_1_-score for GPT-4.1, Gemini 2.5 Pro, and Llama 4 Maverick, but reduced both for Claude 4.5 Sonnet, consistent with the pattern seen in EM accuracy. These results demonstrate that domain-specific retrieval enhances models’ ability to predict ISS within correct categories, even when exact numerical predictions may be imprecise.

**Table 2:** Accuracy and macroaveraged F1-score for classification of Injury Severity Score (ISS) ranges in our physician-verified test set of 100 trauma cases from Chang Gung Memorial Hospital, Linkou, Taiwan, in 2022. Cases were classified as mild (ISS 1-8), moderate (ISS 9-15), severe (ISS 16-24), or profound (ISS 25-75). Original denotes prompting without retrieval-augmented generation (RAG), standard RAG uses the pretrained MedCPT retriever [18], and domain-specific RAG (DS-RAG) uses a retriever fine-tuned on trauma-related ICD-10-CM (International Classification of Diseases, Tenth Revision, Clinical Modification) data. Values indicate the mean accuracy over 3 runs. International Classification of Diseases and R statistical software (ICDPIC-R; gem_max) [24] is a deterministic ICD-10-CM–based tool and is reported as a single reference value without SD.

Metric and method		GPT-4.1, mean (SD)	Gemini 2.5 Pro, mean (SD)	Claude 4.5 Sonnet, mean (SD)	Llama 4 Maverick, mean (SD)	ICDPIC-R	
**Accuracy (%)**							80.00
	Original	66.33 (1.15)	69.00 (4.36)	74.33 (1.53)	59.00 (1.73)		
	Standard RAG	70.67 (1.53)	73.67 (2.52)	67.00 (1.00)	70.33 (2.52)		
	DS-RAG	84.00 (1.00)	76.00 (0.00)	76.00 (2.65)	74.33 (1.53)		
***F*_1_-score (%)**							73.32
	Original	67.37 (1.58)	67.84 (3.68)	73.39 (1.81)	62.72 (2.06)		
	Standard RAG	68.31 (1.73)	75.52 (3.50)	67.29 (1.25)	69.76 (4.09)		
	DS-RAG	84.08 (1.03)	78.39 (0.03)	79.41 (2.71)	74.85 (1.12)		

### Impact of Number of Retrieved References on Performance

This section measures the retrieval performance using different values of *k* (number of retrieved AIS code entries) for RAG. As shown in Table 3, higher recall scores were achieved by increasing *k*, but at the cost of retrieval precision, which fell from 16.15% at *k*=3 to 10.81% at *k*=5 and 7.03% at *k*=10 for DS-RAG. Increasing *k* to 10 raised overall EM accuracy (77.7%) but reduced accuracy on profound cases from 52.4% to 47.6%. This suggests that additional retrieved AIS entries may introduce additional noise, which harms performance on complex cases. Reducing *k* to 3 lowered both overall accuracy (72.7%) and profound accuracy (47.6%). We adopted *k*=5 as the default, prioritizing accuracy on profound cases (52.4% vs 47.6% at *k*=10) over the higher overall accuracy at *k*=10 (77.7% vs 75.3%). We note that this choice was made on the test set itself: the substantial expert annotation effort required for physician-verified labels precluded constructing a separate validation set. Table 3 is accordingly reported as a sensitivity analysis, and our comparative conclusions (DS-RAG vs original and standard RAG) hold at every *k* examined. Proxy-task retrieval performance on *ICD-10-CM* is present in Table B.2 in Multimedia Appendix 1.

To see the upper bound of RAG, we also tested the RAG performance of GPT-4.1 using all the correct AIS information provided to calculate ISS scores. Table 3 shows that GPT-4.1 (Gold) achieved EM accuracies of 81.7% and 73.8% for overall and profound cases. These results suggest that profound discharge diagnoses remain challenging for GPT-4.1, even when the correct AIS information is provided. This highlights the importance of comprehensive context when determining a single AIS description. However, it is sometimes difficult to discern all the details in profound trauma, and the lack of a structured format for discharge diagnosis may lead to deviation from reality. Additionally, we found that LLMs tend to speculate about the etiology behind each diagnosis. Even though it is not an anatomical description of the injury, LLMs try to figure it out with an anatomical etiology and generate a new, incorrect diagnosis (eg, “diffuse brain swelling” or “hypoxic encephalopathy” can result from multiple causes, but LLMs tend to consider them to be consequences of severe head injury).

**Table 3:** Performance comparison for using different numbers (k) of retrieved Abbreviated Injury Scale (AIS) descriptions with GPT-4.1 for automated Injury Severity Score (ISS) calculation in our physician-verified test set of 100 trauma cases from Chang Gung Memorial Hospital, Linkou, Taiwan, in 2022. Precision and recall are macroaveraged retrieval metrics, and exact-match (EM) accuracy requires the predicted ISS to equal the physician-verified ISS. Performance is reported for the complete test set and the profound injury subgroup (ISS 25-75; n=14). Gold denotes an oracle setting in which the ground-truth AIS codes are directly provided to the large language model, bypassing the retriever; its precision and recall are therefore 100%.

Method (using GPT-4.1)	*k*	Precision (macro; %)	Recall (macro; %)	EM accuracy (%), mean (SD)^a^	EM accuracy for profound severity (%), mean (SD)^a^
Gold (Oracle)	N/A^b^	100	100	81.7 (2.5)	73.8 (8.2)
Standard RAG^c^	3	11.90	44.05	60.0 (1.7)	42.9 (7.1)
DS-RAG^d^	3	16.15	55.47	72.7 (2.1)	47.6 (8.2)
Standard RAG	5	9.72	54.88	58.7 (1.5)	42.9 (7.1)
DS-RAG (proposed)	5	10.81	59.47	75.3 (0.6)	52.4 (4.1)
Standard RAG	10	5.41	58.58	62.7 (0.6)	50.0 (0.0)
DS-RAG	10	7.03	71.83	77.7 (1.2)	47.6 (4.1)

^a^Values indicate mean accuracy over 3 runs.

^b^N/A: not applicable.

^c^RAG: retrieval-augmented generation.

^d^DS-RAG: domain-specific retrieval-augmented generation.

### Ablation Study on RefExp

To address the trade-off between recall and precision during retrieval, we introduced a RefExp mechanism. This method augmented the initial retrieval results by including additional AIS descriptions that share the same anatomical prefix (specifically, the first 3 words) with the top-*k* retrieved entries. This approach ensured that semantically related injuries within the same anatomical region were retrieved together, improving macrorecall (53.03% to 59.47%) at the cost of a modest decrease in macroprecision (13.00% to 10.81%; Table 4). This trade-off proved favorable overall: EM accuracy improved from 72.7% to 75.3%, indicating that the generator tolerated the additional candidates while benefiting from the improved coverage.

**Table 4:** Ablation study of reference expansion (RefExp) in the domain-specific retrieval-augmented generation (DS-RAG) framework using GPT-4.1 in a physician-verified test set of 100 trauma cases from Chang Gung Memorial Hospital, Linkou, Taiwan, in 2022. RefExp adds AIS descriptions sharing the same 3-word anatomical prefix as the initially retrieved entries. Precision and recall are macroaveraged retrieval metrics, and exact-match (EM) accuracy requires the predicted ISS to equal the physician-verified ISS. Values in parentheses indicate SD over 3 runs.

Method	Precision (macro; %)	Recall (macro; %)	EM accuracy (%)
DS-RAG (proposed)	10.81	59.47	75.3 (0.6)
DS-RAG without RefExp	13.00	53.03	72.7 (0.6)

### Case Study: Error Analysis

To understand why LLMs make mistakes in ISS calculation, we show a representative failure case using an example discharge diagnosis (Textbox 1) and compare GPT-4.1 outputs across different settings (Table 5). In this case, the discharge diagnosis describes (1) a traumatic brain injury with a large left temporal acute-on-chronic subdural hematoma and midline shift, (2) a left ear upper pole traumatic amputation with primary suture, and (3) a left proximal humerus fracture, followed by several noninjury conditions and underlying diseases (Textbox 1). The ground-truth AIS codes extracted for ISS computation are 140656 (head/neck severity 5), 752600 (extremities severity 2), and 240299 (face severity 1), yielding ISS=30 (Textbox 1).

**Representative deidentified discharge diagnoses selected from the physician-verified test set of trauma cases treated at Chang Gung Memorial Hospital, Linkou, Taiwan, in 2022. The textbox presents the free-text discharge diagnosis and the physician-verified Abbreviated Injury Scale (AIS) codes used to calculate the gold-standard Injury Severity Score of 30. The discharge diagnosis text is reproduced verbatim from the original medical record, including typographical or formatting irregularities.**

**Discharge diagnosis**

1. Traumatic brain injury with left temporal acute on chronic subdural hematoma 1.3 cm with midline shift 2 mm, post left frontotemporal minicraniotomy for subdural hematoma removal with subdural drain placement on April 27, 2022
2. Left ear upper pole traumatic amputation post primary suture for hemostasis on April 27, 2022
3. Left proximal humerus recent fracture under conservative treatment
4. Hypokalemia
5. Insufficient hemoglobin [Underlying diseases]. Left hip fracture post operation. Alzheimer disease. Arrhythmia, under plavix use. Parotid tumor s/p op

**Gold AIS codes**

1. 140656 (Cerebrum, Hematoma, Subdural, Large, Severity 5), 240299 (Ear, NFS, Severity 1), 752600 (Humerus Fracture, NFS, Severity 2)

**Table 5:** Qualitative error analysis of GPT-4.1 predictions for the representative trauma case described in Textbox 1. The case included a large subdural hematoma, traumatic ear amputation, and proximal humerus fracture, with physician-verified Abbreviated Injury Scale (AIS) severities of 5 for the head/neck region, 1 for the face region, and 2 for the extremities, yielding an ISS of 30. Condensed outputs are compared across original prompting without retrieval-augmented generation (RAG), standard RAG, domain-specific RAG (DS-RAG), and Gold RAG using ground-truth AIS codes. The AIS severities and key error patterns shown for each method were taken from the first run. Table C.2 in Multimedia Appendix 1 shows the “explanation” field of each model output. The output logs for this example are provided in Multimedia Appendix 2.

Method	AIS severity			Key error pattern from model output
	Head/neck	Extremities	Face	
Original (without RAG)	4	3	—^a^	Underestimates head injury (AIS 4 vs 5) and overestimates ear; additionally treats hip fracture/medical history as acute injury in ISS computation.
Standard RAG	4	2	2	Retrieval does not help: more irrelevant retrieved information provided. Model mainly relies on discharge diagnosis; still underestimates head injury and overestimates ear.
DS-RAG	4	3	2	Better retrieval: more relevant retrieved information compared with standard RAG; persists in head underestimation and ear overestimation.
Gold RAG (Oracle)	5	2	1	Correct only when ground-truth AIS codes are explicitly provided, yielding ISS=30.

^a^Not available.

We first examine retrieval quality under RAG. Although this case has clear ground-truth AIS labels, both the MedCPT retriever (standard RAG) and our domain-specific retriever return AIS code candidates that do not overlap with the ground-truth codes (Table 5; full retriever predictions are provided in Table C.1 in Multimedia Appendix 1). Importantly, the domain-specific retriever tends to retrieve more clinically relevant candidates with severities closer to the correct injuries (eg, humerus fracture– and ear injury–related entries), whereas MedCPT retrieval is less aligned (Table C.1 in Multimedia Appendix 1). Notably, the MedCPT retriever returned “Decapitation” (AIS severity 6, a maximal injury) as a candidate for this patient, whose actual head-region injuries were a subdural hematoma and an ear amputation. This illustrates the clinical safety implications of semantic drift in general-purpose retrievers: surface-level semantic similarity (head-region trauma involving tissue loss) can surface references whose severity is catastrophically mismatched, and had the generator adopted such a reference, the ISS would have been grossly inflated, misclassifying the patient at the highest severity level. In this case, GPT-4.1 did not adopt the spurious candidate (Table 5), suggesting that the generator provides a degree of robustness against retrieval noise; however, relying on the generator to filter clinically implausible references offers no safety guarantee. This further motivates a domain-specific retriever that returns severity-plausible candidates in the first place.

Table 5 summarizes the model outputs as well. Without RAG (original), GPT-4.1 underestimates the head injury (AIS 4 vs 5) despite the explicit mention of a large subdural hematoma and overestimates the ear injury (AIS 2 vs 1). Standard RAG introduces more irrelevant references, which do not correct the failure pattern and may distract the generator (Table 5; Table C.2 in Multimedia Appendix 1). With DS-RAG, the retrieved candidates are more relevant than standard RAG, but the same major error pattern persists (head underestimation and ear overestimation), again resulting in no correction of ISS (Table 5; Table C.2 in Multimedia Appendix 1). Across the 3 runs, the original, standard RAG, and DS-RAG settings produced ISS values of 25, 20, and 20; 24, 33, and 33; and 29, 29, and 29, respectively. Thus, DS-RAG showed greater consistency for this case, although its prediction remained 1 point below the gold-standard ISS. One plausible explanation is supervision mismatch. Our retriever is fine-tuned with *ICD-10*–based pairs, whereas AIS entries require finer-grained morphological and severity-specific matching, potentially leading to retrieval errors. Finally, Gold RAG (Oracle) produced ISS values of 30, 30, and 35 across the 3 runs when the ground-truth AIS codes were explicitly provided, indicating that generation variability can persist even with correct AIS information (Table 5). Overall, this case suggests that failures under RAG frameworks can arise from both incorrect AIS retrieval and generation variability, and improving retrieval alignment between clinical text and AIS coding descriptions is critical for enabling accurate downstream ISS calculation.

## Discussion

### Principal Results

This study demonstrates that our proposed DS-RAG framework greatly enhances the accuracy of LLMs on ISS calculation from unstructured discharge diagnoses. Our key finding is that grounding LLMs with a retriever fine-tuned on domain-specific *ICD-10-CM* data is crucial for performance. This approach outperformed the standard RAG setup for every model tested and improved substantially on the baseline for 3 of the 4, with the GPT-4.1 model achieving a 75.3% EM accuracy. Notably, even when the correct AIS codes were provided directly (Gold), EM accuracy reached only 81.7%; exact reproduction of the registry ISS from discharge-diagnosis text alone is therefore intrinsically bounded well below 100%.

A qualitative analysis of our error cases reveals an intriguing behavior: LLMs tend to “hallucinate etiology.” As noted in our results, models occasionally inferred traumatic causes for nonspecific conditions (eg, attributing “brain swelling” to injury without explicit evidence). This suggests that while RAG provides necessary context, the inherent generative nature of LLMs can lead to overinterpretation. This highlights a critical direction for future research: balancing the reasoning capabilities of LLMs with strict evidence-grounded constraints to prevent medically plausible but factually unsupported inferences. Furthermore, LLMs struggled to distinguish acute traumatic injuries from preexisting conditions listed under the patient’s medical history. For example, in our case study, GPT-4.1 without RAG incorrectly incorporated a prior hip fracture into the ISS calculation, inflating the severity score. This suggests that explicit structural cues separating current injuries from underlying diseases in discharge diagnoses may be necessary for reliable automated coding.

Beyond EM accuracy, we evaluated the models with the tolerance criterion, which accepts predictions within a clinically acceptable range of the true ISS. Under this criterion, models like Llama 4 and Gemini 2.5 Pro reached accuracies approaching 90% for moderate and severe trauma cases, respectively, suggesting the DS-RAG framework produces reliable estimations for most critical cases. ICDPIC-R, by contrast, gains nothing from the tolerance criterion in the moderate and profound ranges: when the deterministic tool is wrong on complex cases, its predictions fall far outside clinically acceptable bounds. Furthermore, the improved *F*_1_-scores across all models highlight the framework’s ability to correctly stratify patients into severity categories, which is valuable for applications where such stratification matters more than precise ISS values.

### Limitations

Despite these promising results, several limitations must be acknowledged. First, the evaluation was conducted on retrospective data from a single institution and a single year of admissions, which may limit the generalizability of our findings. Second, this study used AIS 1998, as mandated by Taiwan’s National Health Insurance System. Since injury coding standards have since been updated (eg, AIS 2005/2015), the generalizability of our findings to institutions using newer AIS versions warrants further investigation. Third, our model relied solely on discharge diagnoses for text-only scenarios. The exclusion of other clinical data, such as imaging reports and laboratory tests, may limit its accuracy, particularly in complex, multi-injury scenarios. This constraint also significantly increased the difficulty of annotation, since many discharge diagnoses lacked sufficient information to reliably infer AIS codes without additional *ICD-10* codes or imaging data. To ensure each discharge diagnosis contains sufficient clinical detail to infer AIS codes, tremendous manual effort from medical experts is required. Consequently, only 100 cases could be labeled with AIS codes and included in the physician-verified test set. The test set is therefore underpowered to detect small between-setting differences in this feasibility study. In addition, the retrieval depth *k* was selected on the test set rather than on a separate validation set, since constructing one would have required additional physician-verified annotations; although the comparative conclusions are unchanged at every *k* examined, the reported headline accuracy at *k*=5 should be interpreted with this in mind, and the profound-range difference that motivated the choice (52.4% vs 47.6%, n=14) is well within run-to-run variability.

Fourth, our findings do not establish the proportion of the broader trauma population to which this approach would apply. The manual review was concluded once the target test set had been assembled rather than carried through the entire sampled pool, so a portion of the sampled cases was never adjudicated. The study was not designed as a prevalence study of documentation adequacy, and the counts we report should not be read as a population-level estimate of how often discharge-diagnosis text is sufficient for ISS derivation.

Future research should extend validation to larger, multi-institutional datasets with differing documentation practices, explore the integration of multimodal clinical data, and prospectively assess how often discharge-diagnosis text alone is adequate for ISS derivation.

### Comparison With Prior Work

Previous studies, such as those using ICDPIC-R [6] or direct ICD-to-AIS mapping [5], relied on structured administrative data. While these methods offer deterministic outputs, they are often limited by the quality of initial ICD coding and cannot capture the nuances of free-text clinical narratives. Our findings position LLM-based severity scoring as a text-based alternative to deterministic code-based tools. Our LLM-based approach, particularly with DS-RAG, demonstrates the capability to interpret unstructured descriptions directly, potentially bypassing the errors introduced during the initial ICD coding process.

### Implications for Clinical Deployment

ISS is assigned after treatment for registry entry, so an incorrect score affects benchmarking and resource planning rather than the immediate care of the patient concerned; it is nonetheless consequential, and we intend this approach as decision support under clinician supervision rather than autonomous scoring. A practical safeguard would be to withhold the score when the model’s own reasoning suggests it is unreliable. One promising basis for such a checkpoint may already lie in the output format used here: every response must explain how it derived the AIS codes and ISS and must report any limitations and uncertainties. A checkpoint might be defined on this reasoning, so that where a score appears to rest on speculation, or where the model reports that the record lacks the detail needed to assign a severity, the ISS would be withheld and the case referred for manual coding. However, this work focuses on the accuracy of the ISS calculation itself, and we did not evaluate such a checkpoint empirically. Designing and validating a practical trigger is a necessary step toward clinical deployment.

### Conclusions

This study demonstrates the feasibility of using LLMs for automated ISS calculation from unstructured discharge diagnoses. Our key contribution lies in developing a domain-specific retriever training strategy using *ICD-10* trauma codes as a proxy for AIS codes, addressing the lack of labeled AIS data. LLMs with the DS-RAG framework outperformed the standard RAG setup across all tested models and improved on the baseline for 3 of the 4, with GPT-4.1 achieving an EM accuracy of 75.3% and substantial improvements in tolerance-based metrics from mild to severe ISS ranges. This approach demonstrates the potential to reduce manual coding workload and improve data quality in trauma registries.

## Appendix

Multimedia Appendix 1 Supplementary materials.

Multimedia Appendix 2 The complete outputs for the 4 models in the case study (Table 5).

## Abbreviations

AIS: Abbreviated Injury Scale
DS-RAG: domain-specific retrieval-augmented generation
EM: exact-match
ICD: International Classification of Diseases
ICD-10: International Statistical Classification of Diseases, Tenth Revision
ICD-10-CM: International Classification of Diseases, Tenth Revision, Clinical Modification
ICD-10-GM: International Statistical Classification of Diseases and Related Health Problems, Tenth Revision, German Modification
ICD-9-CM: International Classification of Diseases, Ninth Revision, Clinical Modification
ICDPIC-R: International Classification of Diseases and R statistical software
ISS: Injury Severity Score
LLM: large language model
RAG: retrieval-augmented generation
RefExp: reference expansion

## Data Availability

The physician-verified test set analyzed in this study is available in the GitHub repository [25]. The full trauma registry cohort is not publicly available due to the data governance policy of Chang Gung Memorial Hospital.
