# Supplementary material for: Automating Injury Severity Score Calculation From Free-Text Discharge Diagnoses Using Domain-Specific Retrieval-Augmented Large Language Models: Retrospective Validation Study
Source: JMIR Med Inform. 2026 Oct 13;14:e93922. doi: 10.2196/93922 (PMC13714527; doi:10.2196/93922)
Supplement: Supplementary file 1 [file medinform_v14i1e93922_app1.docx]

## Appendix

### A. Prompts

Table A.1. Prompts for the LLMs used in this study. Text in *italics* is used in the RAG settings only. Input discharge summary is placed at {DISCHARGE_SUMMARY_TEXT}, while the retrieved references are placed at {REFERENCE_DOCUMENTS} for RAG.

| System Prompt | You are an experienced trauma registrar responsible for assigning trauma scores based on discharge summary text diagnoses. You must provide a detailed analysis of traumatic injuries, assign AIS (Abbreviated Injury Scale) scores, and calculate the ISS (Injury Severity Score) using the three most severely injured body regions. |
| --- | --- |
| User Prompt | Please analyze the following discharge summary of a trauma patient and provide an estimated Injury Severity Score (ISS):  ISS is a scale to categorize the severity of trauma patients. To calculate the ISS score for each discharge summary, we need to determine the Abbreviated Injury Scale (AIS) score for each injury in each body region. AIS is on a scale of 1 to 6, 1 being a minor injury and 6 being maximal (currently untreatable). The currently used system of AIS is published in 1998 (AIS 1998).  The ISS is calculated by taking the squared sum of the top three highest AIS scores from the six body regions:  1. Head and Neck  2. Face  3. Chest  4. Abdomen  5. Extremities (including pelvis)  6. External (skin)  When multiple injuries occur in the same body region, only the highest AIS score is used. If multiple injuries have the same AIS score, count the region only once.  Example 1:  AIS 1 for fractured mandible (face)  AIS 3 for injured spleen (abdomen)  AIS 4 for injured liver (abdomen)  The ISS score would be 1² + 4² = 17, taking the highest score for the abdomen.  Example 2:  AIS 2 for fractured sternum (chest)  AIS 2 for fractured ribs (chest)  AIS 3 for compression fracture of lumbar vertebrae (abdomen)  Then the ISS score will be 2² + 3² = 13.  Because both injuries are assigned to the same ISS body region, only the highest AIS score from the chest region is used once in the ISS calculation.  In your analysis, please:  1. Identify the most significant traumatic injuries of each region according to the AIS directly based on the text in the discharge summary, **without considering if the patient recovered or has been treated**.  2. Assign AIS scores to each relevant injury based on severity.  3. Determine the three most severely injured body regions.  4. Calculate the ISS by summing the squares of the highest AIS scores from the three most severely injured body regions.  *Consider reference documents where applicable, but prioritize the discharge summary. If reference documents do not match, make a judgment based solely on the discharge summary.*  Provide the analysis in JSON format with these keys:  - "explanation": brief explanation of how AIS scores and ISS were calculated.  - "limitations": optional, mention any limitations.  - "uncertainties": optional, mention any uncertainties.  - "ISS": the calculated ISS score.  *Reference documents:*  *{REFERENCE_DOCUMENTS}*  Discharge summary:  {DISCHARGE_SUMMARY_TEXT} |

### B. Implementation Details

#### B.1. Training the Retriever for RAG

We used the official code provided by Jin et al [18]. for training the domain-specific retriever, using a single RTX 2080 Ti GPU. Additionally, we used the Faiss library to build the vector database for RAG. The retrievers for the Standard and the DS-RAG settings were implemented with PyTorch (version: 2.4.0+cu118) and Hugging Face Transformers (version: 4.39.3).

Following Jin et al. [18], we employed an in-batch negative sampling strategy: for each (discharge diagnosis, positive ICD-10 description) pair, all other diagnoses and descriptions within the batch served as negative samples. The training objective was to maximize the similarity of positive pairs while minimizing it for all negative pairs. Since the semantic relationships between discharge summaries and ICD-10 descriptions are close to those between discharge summaries and AIS code descriptions, the trained retriever is expected to effectively retrieve relevant AIS codes for a given discharge summary.

Due to the absence of AIS-labeled training data, hyperparameters (e.g., number of epochs, learning rate) for the retriever were initially tuned using ICD-10 trauma code retrieval tasks as a surrogate objective. Retrieval performance on this proxy task is reported in Appendix B.4.

#### B.2. Details for the use of LLM APIs

We used the OpenAI and Anthropic APIs to run the GPT-4.1 and Claude 4.5 Sonnet models. For Gemini 2.5 Pro and Llama 4 Maverick, we used Vertex AI in Google Cloud to conduct the experiments. All experiments were run with temperature = 0; nucleus sampling (top-p) was left at each provider's default. No maximum output length was imposed for GPT-4.1, Gemini 2.5 Pro, or Llama 4 Maverick; for Claude, the Anthropic API requires an explicit limit, which was set to 1,000 tokens (no final prediction was affected). GPT-4.1 was called with the JSON-object response format. Because LLM API inference is not strictly deterministic even at temperature 0, each experiment was repeated three times, and we report the mean and standard deviation across runs. The exact model identifiers were gpt-4.1-2025-04-14, gemini-2.5-pro, claude-sonnet-4-5-20250929, and llama-4-maverick-17b-128e-instruct-maas.

#### B.3. Reference Expansion Details

For each AIS 1998 description, commas are removed, and the text is tokenized by whitespace; the anatomical prefix is defined as the first three tokens, compared by exact string match. Descriptions containing fewer than three tokens (e.g., "Ear, NFS") are excluded from grouping and are never expanded. Same-prefix groups are precomputed over the AIS 1998 dictionary, and at retrieval time each top-k code is deterministically expanded to its full group, with duplicates removed; codes not belonging to any group are retained as-is. With k = 5, this expansion increased the average number of reference entries per query from 5 to 9.14 (maximum 60) and the average input prompt length by 136 tokens (854.8 to 990.8 tokens, +15.9%), measured over all GPT-4.1 DS-RAG queries in the test set.

We selected the three-token prefix by comparing the grouping behavior of two-, three-, and four-word prefixes over the full AIS 1998 dictionary; Table B.1 summarizes the comparison. Prefix length trades coverage against specificity. A two-word prefix groups more of the dictionary (1,165 of 1,366 codes) but produces coarse groups, with a mean group size of 6.07 entries and a largest group of 39. Because Reference Expansion adds every member of a matched group to the prompt, coarse groups introduce candidate descriptions whose severity differs substantially from the retrieved entry, and severity is the quantity that determines the ISS. The two-word prefix "Upper Extremity", for example, collapses 33 codes spanning AIS severities 1 through 9 into a single group, which the three-word prefix separates into 12 anatomically distinct groups. In the AIS 1998 dictionary, severity 9 is the coding convention for injuries whose severity cannot be specified; it is a placeholder outside the ordinal 1–6 scale, and no such code appears in the physician-verified test set labels. A four-word prefix produces the tightest groups but covers only 400 codes: 499 descriptions contain fewer than four words (for example, "Ear, NFS") and cannot be grouped at all, and a further 467 have a prefix shared with no other entry, so roughly two-thirds of the dictionary never triggers expansion. The three-word prefix retains coverage of 767 codes while keeping groups anatomically coherent.

Table B.1. Grouping behavior of the AIS 1998 dictionary (1,366 entries) under two-, three-, and four-word anatomical prefixes. Codes grouped is the number of entries belonging to a group of two or more; an entry is not grouped if its description contains fewer words than the prefix length, or if no other entry shares its prefix. Reference Expansion adds candidates only for grouped entries.

| Prefix length | Codes grouped | Mean group size | Largest group |
| --- | --- | --- | --- |
| 2 words | 1,165 | 6.07 | 39 |
| *3 words (adopted)* | *767* | *4.51* | *22* |
| 4 words | 400 | 3.74 | 12 |

#### B.4. Proxy-task retrieval performance

Because no AIS-labeled training data were available, the domain-specific retriever was trained on ICD-10-CM descriptions as a proxy for AIS descriptions (Appendix B.1). To verify that this proxy objective produced a genuinely improved retriever, we evaluated retrieval directly on the proxy task using the physician-verified test set of 100 cases, which was withheld in its entirety from retriever training. Table B.2 reports the result. The domain-specific retriever retrieves the correct ICD-10-CM codes considerably more often than the general-purpose MedCPT retriever, with a macro recall of 29.40% against 4.12%; both are far above the approximately 0.01% expected from random selection among 38,243 candidate codes. This indicates that the gains observed in the end-to-end ISS results are attributable to improved retrieval rather than to incidental differences in the RAG pipeline.

Table B.2. ICD-10-CM proxy-task retrieval performance on the physician-verified test set (n = 100), which was fully withheld from retriever training. For each discharge diagnosis, the top 5 candidates were retrieved from 39,391 trauma-related ICD-10-CM descriptions (S00–T75; 38,243 unique codes); recall is computed with an exact code-match criterion. The expected recall of random top-5 retrieval is approximately 0.01%.

| Retriever | Macro recall (%) | Micro recall (%) |
| --- | --- | --- |
| MedCPT (general-purpose) | 4.12 | 4.39 |
| Domain-specific retriever (ours) | 29.40 | 17.07 |

#### B.5. Implementation Details for Statistical Analysis

All statistical analyses were performed in Python (version 3.11) using the statsmodels package (version 0.14.6; statsmodels.stats.contingency_tables.mcnemar with the exact option, and statsmodels.stats.proportion.proportion_confint with the Wilson method).

### C. Error Analysis

This appendix provides the supplementary data for the case study discussed in Section “Case Study: Error Analysis” of the main text. Table C.1 presents the detailed comparison of retrieved AIS codes between the MedCPT and Domain-Specific retrievers. Table C.2 provides the full input details and the raw JSON outputs generated by GPT-4.1 across different settings.

Table C.1. Ground-truth AIS codes and the ones predicted by different retrievers.

| AIS_ID | AISSEVERITY | AISDESCRIPTION | ISSREGION |
| --- | --- | --- | --- |
| *Gold AIS Codes (ISS=30)* | | | |
| 140656 | 5 | Cerebrum, Hematoma, Subdural, Large | Head or Neck |
| 240299 | 1 | Ear, NFS | Face |
| 752600 | 2 | Humerus Fracture, NFS | Extremities or pelvic girdle |
| *AIS Codes from the MedCPT Retriever (for Standard RAG)* | | | |
| 311000 | 6 | Decapitation | Head or Neck |
| 110402 | 1 | Scalp, Contusion (includes subgaleal hematoma) | External |
| 150200 | 3 | Skull, Fracture, Base | Head or Neck |
| 150202 | 3 | Skull, Fracture, Base, w/o CSF Leak | Head or Neck |
| 150204 | 3 | Skull, Fracture, Base, w/CSF Leak | Head or Neck |
| 150206 | 4 | Skull, Fracture, Base, Complex | Head or Neck |
| 150400 | 2 | Skull, Fracture, Vault, NFS | Head or Neck |
| 150402 | 2 | Skull, Fracture, Vault, Closed | Head or Neck |
| 150404 | 3 | Skull, Fracture, Vault, Comminuted | Head or Neck |
| 150406 | 4 | Skull, Fracture, Vault, Complex - Torn, exposed, or loss brain tissue | Head or Neck |
| 150408 | 4 | Skull, Fracture, Vault, Massively Depressed | Head or Neck |
| *AIS Codes from the Domain-specific Retriever (for DS-RAG)* | | | |
| 240208 | 1 | Ear, Inner or Middle Ear Injury | Face |
| 752604 | 3 | Humerus Fracture, Open/Displaced/Comminuted | Extremities or pelvic girdle |
| 240220 | 1 | Ear, Vestibular Apparatus Injury | Face |
| 740600 | 2 | Upper Extremity, Joint Capsule Laceration | Extremities or pelvic girdle |
| 340202 | 2 | Larynx, Contusion (hematoma) | Head or Neck |

Table C.2. Model outputs using GPT-4.1 for the discharge diagnosis in Table 5 (main text). For readability, only the "explanation" field of each JSON output is shown; the "limitations" and "uncertainties" keys were optional per the prompt. The complete, unedited output logs are provided as a separate supplementary file.

| Model | Explanation |
| --- | --- |
| Original | The discharge summary describes several traumatic injuries. For each, the most severe injury in each body region is assigned an AIS score based on the 1998 AIS manual. The three highest AIS scores from different body regions are squared and summed to calculate the ISS.\n\n1. Head and Neck: The patient has a traumatic brain injury with a 1.3 cm acute on chronic subdural hematoma and 2 mm midline shift, requiring craniotomy. According to AIS 1998, an acute subdural hematoma >1 cm with midline shift is typically AIS 4 (Serious). The left ear upper pole traumatic amputation is also in this region, but the brain injury is more severe (AIS 4 vs. ear amputation, which is usually AIS 2).\n\n2. Face: No significant facial injuries described (ear is considered head/neck in AIS, not face).\n\n3. Chest: No traumatic injuries described.\n\n4. Abdomen: No traumatic injuries described.\n\n5. Extremities (including pelvis): Left proximal humerus fracture (recent, under conservative treatment) and left hip fracture (post operation). Proximal humerus fracture is typically AIS 2. Hip fracture (femoral neck or intertrochanteric) is usually AIS 3 if closed, not open, and not associated with major vascular injury. The higher of these is AIS 3 for the hip fracture.\n\n6. External: No significant external injuries described.\n\nThe three most severely injured regions are: Head and Neck (AIS 4), Extremities (AIS 3), and no other region with a traumatic injury above AIS 1. The ear amputation is less severe than the brain injury and does not count as a separate region for ISS.\n\nISS = 4^2^ (Head/Neck) + 3^2^ (Extremities) = 16 + 9 = 25. |
| Standard RAG | To calculate the ISS, I first identified all traumatic injuries and assigned AIS scores based on the 1998 AIS system and the provided reference documents. The most significant injuries by region are: (1) Head and Neck: Traumatic brain injury with acute on chronic subdural hematoma (SDH) with midline shift, which is a severe injury (AIS 4); (2) Face: Left ear upper pole traumatic amputation, which is a partial auricular amputation (AIS 2); (3) Extremities: Left proximal humerus fracture (AIS 2) and left hip fracture (AIS 2). Only the highest AIS score per region is used. The three most severely injured regions are Head and Neck (AIS 4), Extremities (AIS 2), and Face (AIS 2). The ISS is calculated as 4^2^ + 2^2^ + 2^2^ = 16 + 4 + 4 = 24. |
| DS-RAG | The discharge summary describes several traumatic injuries. Each injury was assigned an AIS score based on the 1998 AIS system and the provided reference documents. The most significant injuries by region are: (1) Head and Neck: Traumatic brain injury with acute on chronic subdural hematoma (SDH) with midline shift, which is a severe injury (AIS 4); (2) Face: Left ear upper pole traumatic amputation, which is a partial ear amputation (AIS 2); (3) Extremities: Left proximal humerus fracture (recent) and left hip fracture (post operation). The humerus fracture is open/displaced/comminuted (AIS 3 per reference), and the hip fracture (proximal femur) is also typically AIS 3. Only the highest AIS in the region is used, so Extremities is AIS 3. The three most severely injured regions are Head/Neck (AIS 4), Extremities (AIS 3), and Face (AIS 2). The ISS is calculated as 4^2^ + 3^2^ + 2^2^ = 16 + 9 + 4 = 29. |
| Gold RAG | The discharge summary describes several traumatic injuries. For each, the most severe injury in each body region is assigned an AIS score based on the 1998 AIS system and the provided reference documents. The three most severely injured body regions are then identified, and their highest AIS scores are squared and summed to calculate the ISS.\n\n1. Head and Neck: The patient has a left temporal acute on chronic subdural hematoma (SDH) measuring 1.3 cm with a 2 mm midline shift. According to the reference, 'Cerebrum, Hematoma, Subdural, Large' is AIS 5. The presence of midline shift, even if small, and the size (>1 cm) supports a severe injury (AIS 5).\n\n2. Face: The patient has a left ear upper pole traumatic amputation. The reference for 'Ear, NFS' is AIS 1. Even though this is an amputation, ear injuries are generally not life-threatening and are scored low in AIS (typically 1).\n\n3. Chest: No traumatic injuries described.\n\n4. Abdomen: No traumatic injuries described.\n\n5. Extremities (including pelvis): The patient has a left proximal humerus fracture (reference: 'Humerus Fracture, NFS' = AIS 2) and a left hip fracture (no specific reference, but hip fractures are typically AIS 2 unless open or with major complications). The highest AIS in this region is 2.\n\n6. External: No significant traumatic skin injuries described.\n\nThe three most severely injured regions are: Head and Neck (AIS 5), Extremities (AIS 2), and Face (AIS 1). The ISS is calculated as 5^2^ + 2^2^ + 1^2^ = 25 + 4 + 1 = 30. |

### D. Examples for Tolerance Thresholds

Table D.1. Examples for the tolerance thresholds. A prediction is accepted only if two conditions both hold: (1) the absolute difference between the square roots of the ground-truth and predicted ISS does not exceed the threshold (1 for the mild ISS group, 0.5 for the remaining severities); and (2) the predicted and ground-truth ISS fall within the same severity interval. Rows rejected by either condition are marked in *italics*.

|  | Ground-truth (GT) | | | | Simulated Model Outputs | | | | Tolerance Threshold:  $\vert\sqrt{\mathrm{ISS}_{G\mathrm{old}}}-\sqrt{\mathrm{ISS}_{\mathrm{Pred}}}\vert$ |
| --- | --- | --- | --- | --- | --- | --- | --- | --- | --- |
|  | AIS Severity | | | $\mathrm{ISS}_{\mathrm{Gold}}$ | AIS Severity | | | $\mathrm{ISS}_{\mathrm{Pred}}$ |  |
| Mild  (1-8) | 1 | - | - | 1 | 2 | - | - | 4 | 1 |
|  | 2 | - | - | 4 | 3 | - | - | 9 | *1* |
|  | 1 | 1 | - | 2 | 2 | 2 | - | 8 | *1.4142* |
| Moderate  (9-15) | 3 | - | - | 9 | 2 | - | - | 4 | *1* |
|  | 1 | 3 | - | 10 | 2 | 3 | - | 13 | 0.4433 |
|  | 1 | 2 | 3 | 14 | 1 | 1 | 3 | 11 | 0.4250 |
|  | 1 | 2 | 3 | 14 | 1 | 1 | 4 | 18 | *0.5010* |
| Severe  (16-24) | 4 | 0 | 0 | 16 | 4 | 2 | - | 20 | 0.4721 |
|  | 4 | 1 | - | 17 | 3 | 3 | 1 | 19 | 0.2358 |
|  | 3 | 3 | 2 | 22 | 3 | 3 | 1 | 19 | 0.3315 |
|  | 3 | 3 | 2 | 22 | 3 | 3 | 3 | 27 | *0.5057* |
| Profound  (25-75) | 4 | 3 | - | 25 | 3 | 3 | - | 18 | *0.7574* |
|  | 4 | 3 | - | 25 | 4 | 2 | - | 20 | *0.5279* |
|  | 5 | 5 | 5 | 75 | 5 | 5 | 4 | 66 | *0.5362* |
|  | 5 | 5 | 3 | 59 | 5 | 5 | 4 | 66 | 0.4429 |

The tolerance thresholds were designed to balance clinical plausibility against inaccuracies. For mild cases (ISS≤8), a threshold of 1 was adopted, as small differences in the square root of ISS correspond to clinically minor variations that are acceptable. However, beyond mild trauma, the absolute ISS values increase rapidly due to the squared formulation. To avoid accepting predictions with excessively large deviations, a stricter threshold of 0.5 was applied for ISS≥9. Examples are in Table D.1.

This design also reflects considerations at the level of individual AIS codes. For injuries with AIS severity below 3, a deviation of one AIS level is clinically tolerable and falls within the intended threshold. By contrast, when the AIS severity reaches 4 or higher, the injuries represent more severe or life-threatening trauma. In such cases, even a single-level deviation may substantially alter the ISS and clinical interpretation, and thus should not be accepted.

### E. Confidence Intervals for Exact-Match Accuracy

Table E.1 reports exact-match accuracy with 95% confidence intervals for all four models under each of the three experimental settings. Accuracies are the mean across three runs, and intervals were computed using the Wilson score interval for a binomial proportion with n = 100, the size of the physician-verified test set. The Wilson interval was preferred over the standard normal approximation because it remains valid for proportions close to 0 or 1 and for samples of this size. Intervals were computed from unrounded mean accuracies.

These intervals characterize the sampling uncertainty that arises from evaluating on a finite test set. They are distinct from the error bars shown in Figures 2-4 (main text), which represent the standard deviation of accuracy across the three repeated runs and therefore describe run-to-run variability rather than sampling uncertainty. Because all settings were evaluated on the same 100 cases, the statistical significance of between-setting differences was assessed using the paired McNemar's test reported in the Results, rather than by inspecting whether these intervals overlap.

Table E.1. Exact-match accuracy with Wilson 95% confidence intervals for all model and setting combinations (n=100 test cases). Values are the mean across three runs; intervals reflect the sampling uncertainty of the test set rather than run-to-run variability.

| Model | Original | Standard RAG | DS-RAG |
| --- | --- | --- | --- |
| GPT-4.1 | 57.0 (47.2–66.3) | 58.7 (48.9–67.8) | 75.3 (66.1–82.7) |
| Gemini 2.5 Pro | 59.0 (49.2–68.1) | 60.3 (50.5–69.4) | 65.3 (55.6–73.9) |
| Claude 4.5 Sonnet | 64.7 (54.9–73.3) | 54.3 (44.6–63.8) | 66.3 (56.6–74.8) |
| Llama 4 Maverick | 47.3 (37.8–57.0) | 55.0 (45.2–64.4) | 62.7 (52.9–71.5) |

### Reference

18. Q. Jin *et al.*, “MedCPT: Contrastive Pre-trained Transformers with large-scale PubMed search logs for zero-shot biomedical information retrieval,” *Bioinformatics*, vol. 39, no. 11, p. btad651, Nov. 2023, doi: 10.1093/bioinformatics/btad651.
